# Solitary fibrous tumor of the lung: diagnostic challenges and surgical management

**DOI:** 10.37349/etat.2026.1002367

**Published:** 2026-04-21

**Authors:** Vasileios Leivaditis, Konstantinos Grapatsas, Francesk Mulita, Sofoklis Mitsos, Efstratios Koletsis, Athanasios Papatriantafyllou, Elias Liolis, Admir Mulita, Periklis Tomos, Manfred Dahm

**Affiliations:** IRCCS Istituto Romagnolo per lo Studio dei Tumori (IRST) “Dino Amadori”, Italy; ^1^Department of Cardiothoracic and Vascular Surgery, Westpfalz Klinikum, 67655 Kaiserslautern, Germany; ^2^Department of Thoracic Surgery and Thoracic Endoscopy, Ruhrlandklinik, West German Lung Center, University Hospital Essen, University Duisburg-Essen, 45269 Essen, Germany; ^3^Department of General Surgery, General University Hospital of Patras, 26504 Patras, Greece; ^4^Department of Thoracic Surgery, Attikon General Hospital, National and Kapodistrian University of Athens, 12462 Athens, Greece; ^5^Department of Cardiothoracic Surgery, General University Hospital of Patras, 26504 Patras, Greece; ^6^Department of Oncology, General University Hospital of Patras, 26504 Patras, Greece; ^7^Department of Medical Physics, General University Hospital of Alexandroupoli, 68100 Alexandroupoli, Greece

**Keywords:** solitary fibrous tumor, lung neoplasm, intraparenchymal tumor, STAT6 immunohistochemistry, surgical resection

## Abstract

Solitary fibrous tumors (SFTs) are rare mesenchymal neoplasms that typically arise from the pleura but may occur in various extrathoracic sites. Primary intraparenchymal pulmonary SFTs without pleural attachment are exceptionally uncommon and often pose diagnostic and therapeutic challenges. We report the case of a middle-aged female patient presenting with progressive dyspnea and a large mass in the left lower lobe on imaging. Computed tomography revealed a well-circumscribed, hypervascular mass occupying the left lower lobe. Bronchoscopic and percutaneous biopsies were nondiagnostic, and surgical resection was pursued. Intraoperatively, the tumor was found to arise from the lung parenchyma without pleural involvement. Histopathological examination demonstrated a spindle-cell neoplasm with the typical “patternless pattern,” and immunohistochemistry confirmed nuclear STAT6 positivity, establishing the diagnosis of SFT. The postoperative course was uneventful apart from a transient pulmonary embolism, which was successfully treated. The patient was discharged in good condition and is under regular radiologic surveillance. SFTs of the lung are rare and often mimic more common pulmonary tumors radiologically. Histologic confirmation with STAT6 immunohistochemistry is crucial for accurate diagnosis. Complete surgical excision remains the mainstay of treatment. Given the risk of late recurrence—especially in large tumors—long-term imaging follow-up is mandatory. This case highlights the importance of considering SFT in the differential diagnosis of large pulmonary masses, the critical role of STAT6-based histopathologic confirmation, and the necessity for prolonged surveillance even after complete resection.

## Introduction

Solitary fibrous tumors (SFTs) are uncommon mesenchymal neoplasms that can arise in virtually any anatomical location [1]. First described in the pleura, they have since been reported in numerous extrathoracic sites, including the lung parenchyma [2].

In the thoracic cavity, most SFTs originate from the visceral or parietal pleura; purely intraparenchymal pulmonary SFTs without pleural attachment are exceedingly rare [3]. Their rarity and nonspecific radiologic appearance often challenge preoperative diagnosis, as they may mimic more common primary lung tumors or benign masses [4].

Although the majority of SFTs follow an indolent course, some exhibit aggressive features such as local recurrence, malignant transformation, or distant metastasis [4]. Prognostic factors, including tumor size, mitotic index, necrosis, and infiltrative growth, have been linked to unfavorable outcomes. The discovery of the NAB2-signal transducer and activator of transcription 6 (STAT6) fusion has advanced diagnostic accuracy, and nuclear STAT6 immunostaining is now considered a sensitive and specific marker for SFT [4, 5].

Complete surgical resection with negative margins remains the cornerstone of treatment because SFTs generally show limited response to chemotherapy or radiotherapy [6]. Long-term follow-up is recommended, as late recurrences or metastases may occur even in histologically benign cases [2, 6].

We report a case of a large SFT arising from the lung parenchyma and discuss the diagnostic and perioperative challenges in the context of current literature.

## Timeline

The timeline is listed in Table 1.

**Table 1:** **Chronological timeline of clinical events in the present case**.

**Time point**	**Clinical events**
4 years before surgery	CT-guided biopsy diagnosed a solitary fibrous tumor (SFT) of the left lower lobe; surgery was initially deferred.
Follow-up period	Progressive tumor growth observed on imaging.
Preoperative evaluation	Progressive exertional dyspnea; CT revealed a large pulmonary mass measuring approximately 15 × 14.5 × 12.5 cm. The multidisciplinary tumor board recommended surgical resection.
Preoperative workup	Pulmonary function testing, bronchoscopy, echocardiography, and abdominal imaging were performed; pancreatic cystic lesions consistent with side-branch IPMN were detected without worrisome features.
Day of surgery	Open left lower lobectomy with mediastinal lymphadenectomy performed.
Postoperative day 0	Initial extubation following surgery.
Early postoperative period	Cardiopulmonary instability requiring re-intubation and cardiopulmonary resuscitation.
Postoperative day 5	Successful extubation following stabilization.
Postoperative imaging	CT revealed a small peripheral pulmonary embolism; anticoagulation therapy was initiated.
Postoperative day 12	Transfer from intensive care to the surgical ward.
Postoperative day 16	Patient discharged in stable condition.
3-month follow-up	Clinical improvement with no evidence of recurrence or metastasis on CT imaging.

CT: computed tomography; IPMN: intraductal papillary mucinous neoplasm.

## Narrative

**Figure 1:**
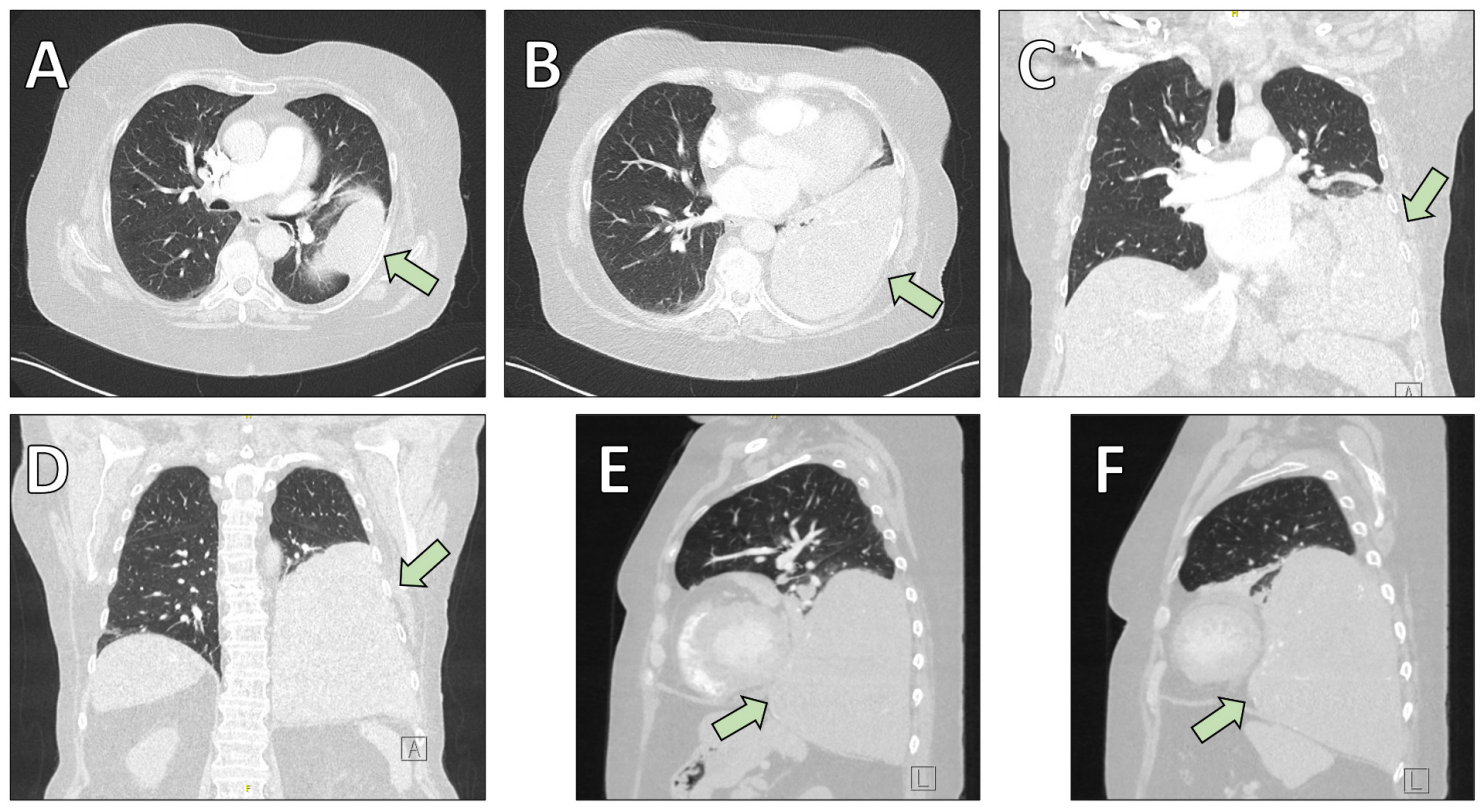
**Preoperative chest computed tomography (CT) illustrating the anatomical localization of the solitary fibrous tumor (arrows).** (**A**, **B**) Axial (transversal) images demonstrating the mass infiltrating the lung parenchyma of the left lower lobe. (**C**, **D**) Coronal reconstructions highlighting the cranio-caudal extension of the lesion. (**E**, **F**) Sagittal reconstructions further delineate tumor size and spatial relationship to adjacent thoracic structures.

**Figure 2:**
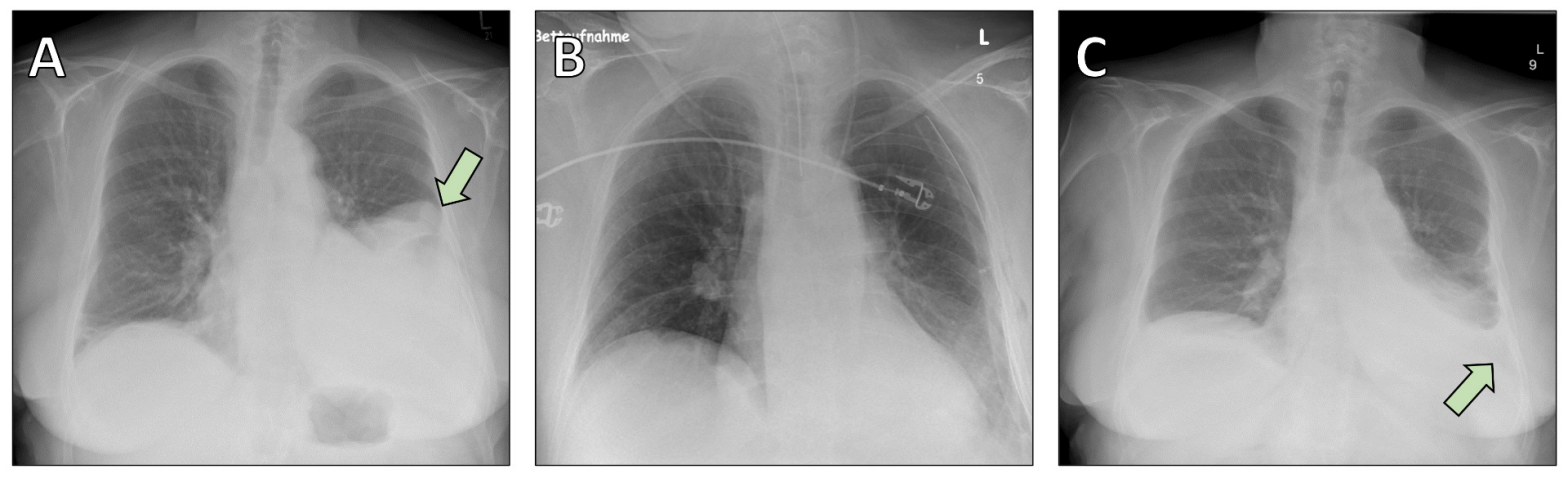
**Chest radiographs obtained during the perioperative period.** (**A**) Preoperative chest X-ray showing a large opacity corresponding to the solitary fibrous tumor in the left lower lobe (arrow). (**B**) Postoperative chest X-ray demonstrating satisfactory re-expansion of the remaining lung parenchyma following left lower lobectomy. (**C**) Postoperative image revealing a pleural effusion (arrow), which was successfully managed with conservative treatment using diuretics.

A 69-year-old woman with a history of pustular psoriasis on long-term corticosteroid therapy and a 20-pack-year smoking history presented with progressive exertional dyspnea. An SFT of the left lower lobe had been diagnosed by computed tomography (CT)-guided biopsy four years earlier, but surgery was initially deferred. Follow-up imaging showed marked tumor growth to approximately 15 × 14.5 × 12.5 cm (Figure 1, Figure 2A), leading the multidisciplinary tumor board to recommend resection.

On examination, she was overweight but in good general condition, with mild expiratory wheezing. Pulmonary function revealed forced expiratory volume in one second (FEV_1_) of 1.17 L (46% predicted), but stair-climb and six-minute walk tests indicated sufficient functional reserve. Echocardiography showed preserved biventricular function with only trace mitral regurgitation. Preoperative bronchoscopy found no endobronchial involvement. Abdominal imaging identified multiple cystic pancreatic lesions consistent with side-branch intraductal papillary mucinous neoplasm (IPMN) without worrisome features. Endoscopic ultrasound confirmed the benign appearance of these lesions, and upper gastrointestinal endoscopy revealed only inflammatory and metaplastic changes without evidence of malignancy. The gastroenterology team recommended no intervention but follow-up imaging and carbohydrate antigen (CA) 19-9 and carcinoembryonic antigen (CEA) measurements in six months.

Given the substantial tumor size and the anticipated risk of intraoperative respiratory and hemodynamic compromise, detailed preoperative anesthetic planning was undertaken in collaboration with the cardiothoracic anesthesia team. Particular attention was paid to the possibility of impaired ventilation following induction due to mass effect and mediastinal shift. Double-lumen endotracheal intubation was performed to allow single-lung ventilation and optimize surgical exposure. Invasive hemodynamic monitoring, including arterial and central venous catheterization, was established prior to incision.

Because large thoracic SFTs may be associated with sudden cardiovascular instability or difficulty in maintaining adequate oxygenation, extracorporeal membrane oxygenation (ECMO) backup was discussed preoperatively with the perfusion team. Although ECMO was not ultimately required in this case, equipment and personnel were kept on standby to allow rapid deployment if severe intraoperative hypoxemia or hemodynamic collapse occurred.

Anesthetic induction and maintenance were conducted with careful titration to minimize cardiovascular depression, and close communication between the surgical and anesthesia teams was maintained throughout the procedure.

**Figure 3:**
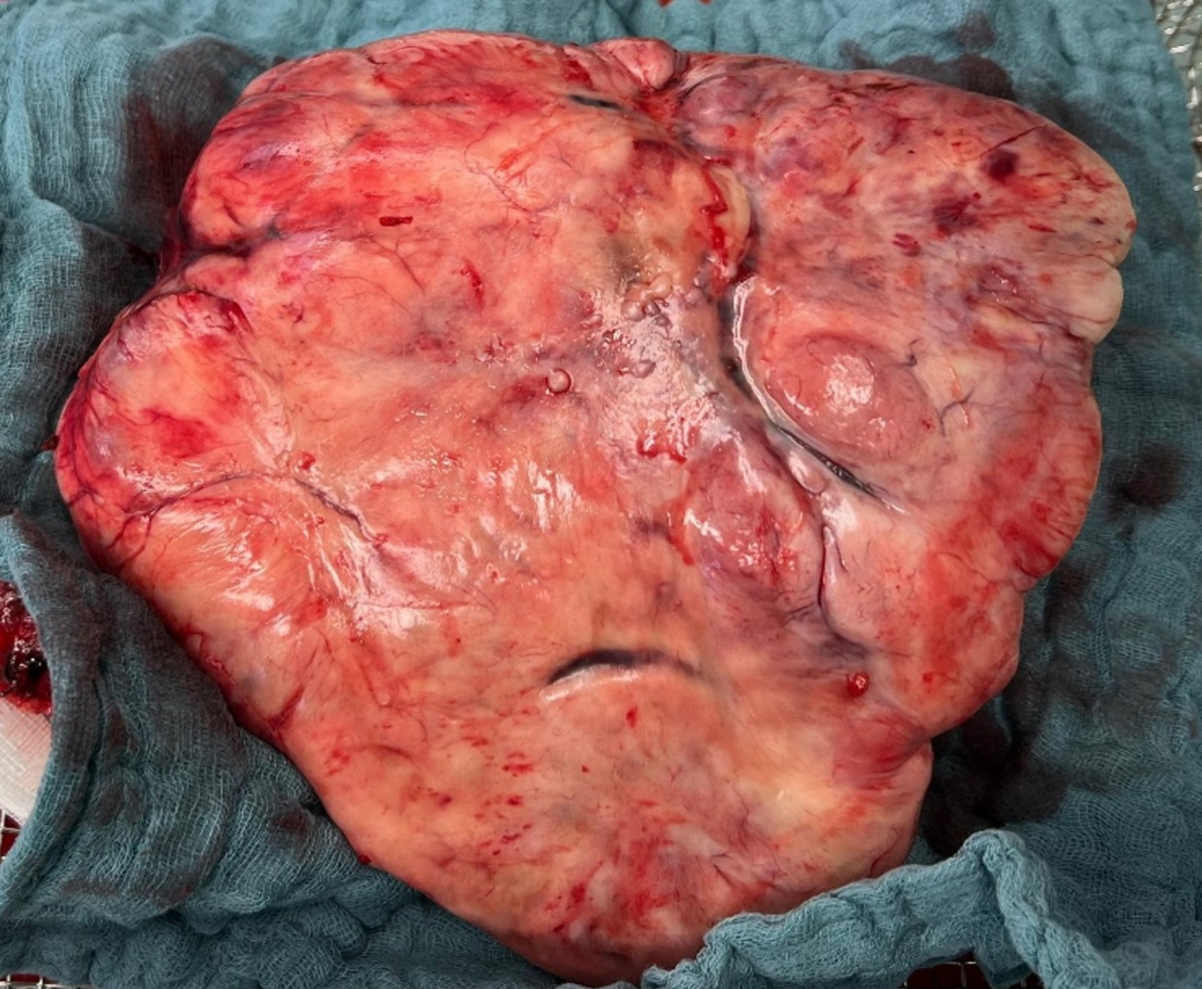
Intraoperative photograph of the resected solitary fibrous tumor following complete surgical excision, illustrating its well-demarcated margins and macroscopic appearance.

**Figure 4:**
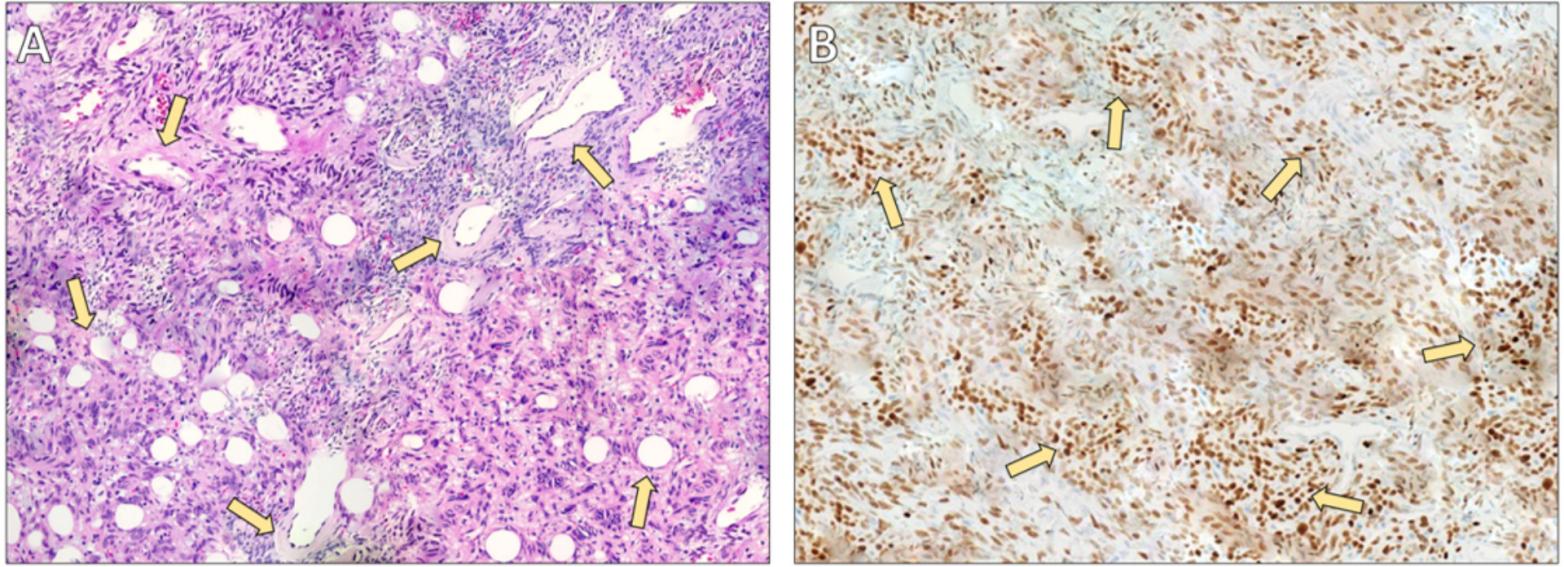
**Histopathological and immunohistochemical findings of the solitary fibrous tumor.** (**A**) Hematoxylin and eosin staining (×40) demonstrating characteristic features of a solitary fibrous tumor, including prominent perivascular hyalinization. Medium- to large-sized vessels are surrounded by thick, concentrically arranged collagenous deposits (arrows). The dense collagen extends from the vascular walls into the surrounding interstitium, creating the typical architectural pattern associated with solitary fibrous tumors. (**B**) Immunohistochemical staining for signal transducer and activator of transcription 6 (STAT6) (×40) showing diffuse and strong nuclear expression in the majority of tumor cells (arrows), confirming the diagnosis. Vascular endothelial cells and surrounding non-neoplastic elements are negative, serving as internal controls.

The patient underwent open left lower lobectomy with mediastinal lymphadenectomy (Figure 2B, Figure 3). She was extubated on the day of surgery but developed cardiopulmonary instability requiring re-intubation and cardiopulmonary resuscitation. She recovered with supportive care and was successfully extubated on postoperative day 5. A postoperative CT scan revealed a small peripheral pulmonary embolism in the right upper lobe artery; anticoagulation with apixaban was started. Postoperative histology confirmed the diagnosis of SFT (Figure 4).

Her subsequent course was marked by transient tachyarrhythmia managed with digoxin, effective diuresis, and empiric antibiotics for elevated inflammatory markers. Thoracic drains were removed uneventfully, and she was transferred to the surgical ward on postoperative day 12. She reported moderate chest pain attributed to resuscitation efforts, but cardiac ischemia was excluded. A postoperative pleural effusion (Figure 2C) was treated with diuretics.

The case was discussed both preoperatively and postoperatively in our institutional multidisciplinary thoracic oncology tumor board, which includes thoracic surgeons, medical oncologists, pulmonologists, radiologists, pathologists, radiation oncologists, and nuclear medicine specialists. Postoperative histopathological examination revealed an 18 cm SFT weighing 1,466 g, with focally increased mitotic activity, hypercellularity, and areas of tumor necrosis. Based on established risk stratification models, these features classified the tumor as high risk for recurrence and metastatic potential.

After a comprehensive review of the current literature and consideration of the complete (R0) resection status, the tumor board recommended structured long-term surveillance rather than adjuvant chemotherapy. This decision was based on the limited and inconclusive evidence supporting adjuvant systemic therapy in completely resected SFTs, even in high-risk cases. A follow-up strategy with regular cross-sectional imaging was therefore initiated, with planned surveillance for at least 10 years.

The remainder of her recovery was uneventful, and she was discharged on postoperative day 16 in stable condition with instructions for ongoing respiratory physiotherapy and laboratory follow-up. At the 3-month follow-up visit, the patient was in good general condition and reported marked improvement in dyspnea compared to her preoperative status. Physical examination was unremarkable. Follow-up contrast-enhanced chest CT showed no evidence of local recurrence, residual disease, or distant metastasis. Anticoagulation therapy for the postoperative pulmonary embolism was continued as planned, without complications. The patient remains under structured long-term radiologic surveillance according to tumor board recommendations.

## Diagnostics

The diagnostic tests and findings are listed in Table 2.

**Table 2:** **Diagnostic tests and findings in the present case**.

**Diagnostic test**	**Findings**	**Interpretation**
Chest CT	Large hypervascular mass in the left lower lobe (15 × 14.5 × 12.5 cm)	Suspicious of SFT
CT-guided biopsy	Spindle-cell neoplasm	Suggestive of SFT
Bronchoscopy	No endobronchial involvement	Non-diagnostic
Pulmonary function test	FEV_1_ 1.17 L (46% predicted)	Reduced pulmonary reserve
Echocardiography	Preserved biventricular function	Suitable for surgery
Histopathology	Spindle-cell tumor with increased mitotic activity and necrosis	Consistent with SFT
Immunohistochemistry	Nuclear STAT6 positivity	Diagnostic confirmation of SFT

CT: computed tomography; SFT: solitary fibrous tumor; FEV_1_: forced expiratory volume in one second; STAT6: signal transducer and activator of transcription 6.

## Patient perspective

Following recovery from surgery, the patient reported a significant improvement in breathing and exercise tolerance compared with her preoperative condition. She expressed satisfaction with the surgical outcome and the multidisciplinary care provided. The patient agreed with the recommended long-term follow-up strategy and continues to participate in regular surveillance imaging.

## Discussion

SFTs are uncommon mesenchymal neoplasms that most frequently arise from the pleura but may occur virtually anywhere in the body, including the lung parenchyma [1–3, 6, 7]. Intraparenchymal pulmonary SFTs without pleural attachment are particularly rare and may be radiologically indistinguishable from other benign or malignant pulmonary masses [6, 7].

### Diagnostic considerations

Histologically, SFTs display a so-called “patternless pattern” of spindle cells with alternating hyper and hypocellular areas and prominent staghorn-type vasculature [6, 8]. The discovery of the *NAB2-STAT6* gene fusion [5] and the resulting nuclear STAT6 immunohistochemical staining [4, 9] has markedly improved diagnostic specificity, allowing SFTs to be distinguished from morphologic mimics such as synovial sarcoma or fibrosarcoma.

### Prognostic factors

While many SFTs behave in an indolent fashion, a significant subset exhibits aggressive behavior with local recurrence or distant metastasis despite apparently complete resection [1, 6, 10, 11]. Several studies have identified tumor size, mitotic index, necrosis, cellularity, and patient age as adverse prognostic indicators [10, 11]. The risk-stratification calculator proposed by the French Sarcoma Group [10] and the Demicco model [11] are widely used tools to estimate recurrence risk. In particular, tumors > 10 cm, as in our patient, have been consistently linked to poorer outcomes [11].

### Surgical treatment and recurrence

Complete surgical excision with negative margins remains the cornerstone of curative therapy [2, 6, 12]. Incomplete resection or capsular disruption increases recurrence risk, emphasizing the importance of meticulous operative technique [12, 13].

Resection of large intrathoracic SFTs may present several intraoperative challenges that warrant careful preoperative planning and surgical vigilance. Dense adhesions to adjacent lung parenchyma, diaphragm, pericardium, or mediastinal structures have been frequently described, particularly in long-standing or large tumors, and may increase the risk of capsular disruption or incomplete excision [14]. Furthermore, SFTs are often markedly hypervascular, receiving blood supply from bronchial, intercostal, internal mammary, or even phrenic arterial branches. Identification and secure ligation of feeding vessels early during dissection is essential to minimize intraoperative blood loss, which can occasionally be substantial [15, 16]. In selected cases, preoperative angiography and embolization have been proposed to reduce vascularity and facilitate safer resection, particularly for giant tumors or when imaging suggests a prominent arterial supply [17].

Vascular involvement of adjacent structures, although uncommon, may necessitate en bloc resection of adherent tissues to achieve negative margins [12, 14, 17]. Surgeons must also remain alert to the possibility of unexpected findings such as occult invasion, tumor friability, or intraoperative hemodynamic instability related to mass effect or manipulation. These factors underscore the importance of experienced thoracic surgical teams and multidisciplinary perioperative coordination when managing giant SFTs.

Reported recurrence rates vary between 3% in histologically benign tumors and more than 20% in those with adverse features [6, 10–13]. When technically feasible, repeat surgical resection of recurrent disease can provide long-term disease control [12].

### Adjuvant and targeted therapies

Conventional chemotherapy and radiotherapy have limited efficacy in SFT and are generally reserved for unresectable or progressive disease [6, 18, 19]. Advances in understanding the angiogenic biology of SFT have led to the successful introduction of anti-angiogenic therapies, such as pazopanib, sunitinib, sorafenib, and bevacizumab, which have shown activity in advanced or metastatic cases [18–21]. Sunitinib in particular has demonstrated durable disease stabilization in several series [20, 21].

### Unique aspects of the present case

Our case is notable for the rare intrapulmonary location, the large tumor size, and the occurrence of a postoperative pulmonary embolism. Although the patient did not develop Doege-Potter syndrome—hypoglycemia due to ectopic insulin-like growth factor 2 (IGF-2) secretion that may occur in large SFTs—awareness of this paraneoplastic phenomenon is important [6]. The substantial tumor growth between the initial diagnosis and eventual surgery underlines the need for timely resection whenever feasible.

### Follow-up recommendations

Because late recurrences—sometimes more than a decade after resection—are well documented, long-term surveillance with periodic chest imaging is essential [2, 6, 10–13]. For tumors with high-risk features (e.g., size > 10 cm, elevated mitotic index, necrosis), follow-up every 6–12 months in the first few years and annually thereafter is advisable.

## Conclusions

SFTs of the lung are rare and often present a diagnostic challenge due to their nonspecific clinical and radiologic features. Histopathologic confirmation with STAT6 immunohistochemistry remains essential for accurate diagnosis. Complete surgical resection with negative margins is the mainstay of treatment and offers the best chance for long-term disease control. However, because tumor size and other histologic factors can predict recurrence, long-term imaging surveillance is mandatory even for apparently benign lesions. Our case highlights the importance of early recognition, prompt surgical intervention, and multidisciplinary management to achieve optimal outcomes in these uncommon tumors.

## Abbreviations

CT: computed tomography
ECMO: extracorporeal membrane oxygenation
SFTs: solitary fibrous tumors
STAT6: signal transducer and activator of transcription 6

## Declarations

### Availability of data and materials

The patient’s data is available through the General University Hospital of Patras electronic medical records. More information can be obtained by contacting the corresponding author.

### Copyright

© The Author(s) 2026.
